# Should we reconsider first-line treatments for glaucoma in the setting of meibomian gland dysfunction and ocular surface disease: Glaucoma treatments and its effects

**DOI:** 10.3389/fopht.2023.958955

**Published:** 2023-02-06

**Authors:** Annie Nguyen

**Affiliations:** Keck School of Medicine, University of Southern California, Roski Eye Institute, Los Angeles, CA, United States

**Keywords:** glaucoma, meibomian gland dysfunction, ocular surface disease, MIGS, SLT

## Introduction

Glaucoma, an optic neuropathy that leads to progressive vision loss, is a leading cause of irreversible blindness globally in patients over forty years old, with more than three million Americans and around eighty million people worldwide affected by this disease, with open angle glaucoma (OAG) being the most common form (1). Ocular hypertension, a risk factor for development of glaucoma, is a condition that occurs when there is elevated intraocular pressure without optic nerve damage, with another three to six million Americans affected by this condition (2). Although there is currently no cure for glaucoma, reduction of intraocular pressure has been shown to slow down progression of the disease (3). Currently, the standard first-line treatment for OAG and ocular hypertension is chronic topical pharmacological management that target intraocular pressure lowering mechanisms (4). In the U.S., classes of topical therapy include the following: prostaglandin analogs (PGA), beta blockers, alpha agonists, carbonic anhydrase inhibitors (CAI), rho-kinase inhibitors, combination formulations, and cholinergic agonists. PGA and beta-blockers are common first-line medications due to excellent daily dosing efficacy and favorable systemic safety profiles. However, oftentimes, more than one eye drop is necessary to achieve adequate IOP-lowering effects, and ocular side effects are major shortcomings (5).

Ocular surface disease (OSD), including meibomian gland dysfunction (MGD), is a common notable debilitating problem that affects many patients receiving long-term topical therapy for glaucoma treatment (6). It causes significant morbidity and has a negative impact on treatment compliance, and quality of life (7). Meibomian glands (MGs) are sebaceous glands present in the tarsus of the upper and lower eyelids and secrete lipids that form the superficial layer of the tear film, essential in maintenance of the ocular surface. MGD is a chronic, diffuse, inflammatory abnormality of the MGs, commonly characterized by terminal duct obstruction and/or qualitative/quantitative changes in the glandular secretion, as defined by the International Workshop on Meibomian Gland Dysfunction (8). The prevalence of MGD in the normal population reported in the literature is highly variable and ranges from 3.5 to 70% and is one of the most common cause of dry eye and OSD (9). Severe MGD can lead to irreversible MG atrophy and chronic dry eye, with the impact of severe dry eye comparable to that of moderate to severe angina according to studies on quality of life impact of dry eye disease (10). With both glaucoma and MGD affecting the aging population and leading to irreversible damage, it is critical to appropriately address both conditions.

## Recent findings on the use of glaucoma medications on meibomian glands

According to several studies, MGD is highly associated with use of IOP-lowering eyedrops, a negative inflammatory effect resulting from the active component, preservatives (particularly and most used, benzalkonium chloride or BAK), or both. In a 3-year longitudinal cohor study, Ambaw et al. found that after trabeculectomy, there was reduction of pro-inflammatory lipid mediators in tears. Soriano et al. reported that glaucoma topical treatments produce MGD, altering their structure and function (11). Kim et al. found that the prevalence of MGD was 82% in the group using topical glaucoma medications and 52.5% in the control group without glaucoma (12). In addition, Arita et al. found that long-term anti-glaucoma eye drop use affects MG morphology and function, with similar MG dropout in PGA and beta-blocker treated eyes (13). Cho and colleagues reported that patients with a higher burden of glaucoma agents had more unstable tear films and more severe MG dropout, with a sub-analysis supporting PGA having more effect on MG dropout than other types of eye drops (14). Mocan et al. found that 92% of patients on topical PGA monotherapy compared with 58% using a non-PGA medication had some signs of MGD (15). Ha et al. compared the effect of preservative-containing (PC) and preservative-free (PF) PGA formulations MG in patients with OAG and found that although both PC and PF formulations can cause damage to the MG in patients using PGA, PC formulations induced more ocular discomfort and more severe MG loss compared to PF formulations (16). Agnifili and colleagues reported that PGA/beta-blocker fixed combinations were less toxic towards MGs and goblet cells compared with the PGA (latanoprost) and beta-blocker (timolol) unfixed combination, with preservative-free-fixed combinations presenting the most tolerated profile (17). Similarly, Lee et al. found that longer duration of use and use of preservative-containing formulation of tafluprost were correlated with more negative effects on MGs (18). Konstas et al. discussed in a critical review the safety and tolerability using topical preservative-free agents in treatment of glaucoma (19).

As outlined above, there is an abundance of literature supporting pharmacological glaucoma therapy causes OSD. Interestingly but not surprisingly, severe OSD has been associated with poor medication compliance and IOP control. Lee and colleagues found that MGD can be an important clinical finding correlated with poor PGA monotherapy compliance in patients with normal tension glaucoma (20). In addition, Batra et al. described in a case series of four patients with inadequately controlled OAG and OSD the impact that OSD management can have on glaucoma outcomes: treating the OSD led to improved IOP control and forestalling the need for surgical intervention in these patients during the study period (21). Moreover, Boso and team found in their case series 74% of patients reported severe symptoms of dry eye disease with 50% of patients having tear film instability and 24% with MGD and that after ocular surface treatment, there was significant improvement of best correct visual acuity, dry eye symptoms, as well as mean IOP from baseline (22).

## Recent findings on alternative first-line therapies for OAG and ocular hypertension

As aforementioned, OAG and ocular hypertension are habitually treated with standard first-line eye drops. However, due to its common side effects on the ocular surface, which can lead to poor compliance and glaucoma management outcomes, alternative first-line therapies should be considered. In 2019, the laser in glaucoma and ocular hypertension (LiGHT) trial, a pivotal study, was published, aimed to compare selective laser trabeculoplasty (SLT) and eye drops to treat patients with OAG and ocular hypertension through a multicentered, observer-masked, randomized controlled trial, where 356 eyes were treated with SLT and 362 eyes were treated with eye drops (23). Compared to the eye drops group at 3 years, primary outcome questionnaire regarding health-related quality of life showed no difference, with also comparable secondary endpoints of visual acuity, IOP and visual field loss mean deviation. Eyes in the SLT group (93%) achieved IOP within target at more visits than in the eye drop group (91%). In addition, none of the eyes in the SLT group required glaucoma surgery compared to 11 eyes in the eye drop group by the end of the study. At the conclusion, 74% of SLT-treated eyes were successfully controlled without additional eye drops for at least 3 years after starting treatment. In addition, there was a 97% probability of SLT as first-line treatment being more cost-effective than eye drops in the UK healthcare setting where the study was based. Given good clinical outcomes, cost-effectiveness, and drop freedom, SLT should be considered as first-line therapy, especially in patients with OSD and MGD.

In patients who are poor candidates for SLT and eye drop use remains a necessity, preservative-free topical glaucoma medications should be considered, especially in the setting of OSD and MGD. In clinical studies, OSD signs and symptoms are consistently better with preservative-free versus preserved formulations without compromising IOP control (24). Manufacturers are increasing their focus on production of preservative-free eye drops or BAK-alternatives and combination therapies aimed at abating exposure to harmful preservatives. Rouland et al. compared the efficacy and safety of preservative-free (PF) latanoprost to BAK-preserved latanoprost in ocular hypertension and primary OAG patients through an international, randomized controlled trial and showed that the PF-formulation had the same efficacy but better local tolerance than the BAK-containing PGA (25). Diminution of the corneal basal nerve density and decreased corneal sensitivity have also been reported from use of BAK-containing eye drops (26). Alternatives for BAK with better side effect profiles are currently available and include the following: SofZia (Alcon Laboratories, Fort Worth, TX), Polyquad (Alcon), and Purite (Allergan, an Abbvie company, Chicago, IL).

Until recent years, glaucoma surgery was unappealing due to relatively higher risk profiles compared to medical therapy. With traditional filtering surgery, risk of infection, inflammation, hypotony, and bleb-related complications are major concerns. More recently, the use of minimally invasive glaucoma surgeries (MIGS) has gained a growing role in glaucoma management given its good safety profile and speedy recovery (27). MIGS could be considered in patients with mild to moderate glaucoma and/or those who are intolerant or noncompliant with standard medical therapy especially in those with OSD. Multiple studies have shown that MIGS with or with concurrent cataract surgery leads to better IOP control and decreased eye drop dependence (28, 29). Looking more specifically at the ocular surface, Schweitzer and colleagues through their prospective, single-arm clinical trial of 47 eyes showed that implantation of trabecular microbypass stents with cataract surgery led to improved IOP and medication reductions and improved ocular surface health (30). Tong et al. reported their findings on impression of conjunctiva showing reduced expression of inflammatory genes, improved conjunctival staining score and decreased tear osmolarity where none of the patients post trabeculectomy remained on anti-glaucoma eye drops 3 years after trabeculectomy (31). Baiocchi et al. assessed the quality of the ocular surface by *in vivo* scanning laser confocal microscopy in POAG treated with Xen 45 Gel Stent, medical therapy, and trabeculectomy and found that ocular surface inflammation was most notable in topical therapy followed by trabeculectomy followed by Xen 45 Gel stents (32). However, it is difficult to comment on the effect of individual factors in cross sectional studies.

## Discussion

In this opinion article, it is the author’s preference and recommendation in the setting of pre-existing OSD and MGD, that those ocular comorbidities be considered when deciding on first-line therapy for new treatment or continued treatment for glaucoma and ocular hypertension. Given glaucoma is a chronic, progressive disease where decades of IOP control is necessary, care must be taken to avoid trading one problem for another and when both problems are present, both glaucoma and ocular surface disease should be addressed. Careful examination and recognition of OSD including MGD is critical. Several studies as mentioned above demonstrate the harmful side effects of topical medical therapy on the ocular surface, both from the active component of the drug and its preservatives. Avoidance of common first-line glaucoma medications such as PGA in those with OSD and MGD should be cogitated. Paradigm shifts such as SLT when appropriate as first-line therapy given its good safety profile and efficacy as well as cost-effectiveness will transform and improve glaucoma care. In addition, consideration of MIGS should be made for those with mild to moderate glaucoma on one or more eye drops with signs of OSD to avoid further issue.

## Author contributions

The author confirms being the sole contributor of this work and has approved it for publication.
